# Flexible video endoscope versus Macintosh laryngoscope for orotracheal tracheal intubation in the lateral position: a study protocol for a randomized controlled trial

**DOI:** 10.1186/s13063-019-3263-1

**Published:** 2019-03-15

**Authors:** Youguang Gao, Bo Lin, Jinghao Huang, Xianzhong Lin, Caizhu Lin

**Affiliations:** Department of Anesthesiology, The First Affiliated Hospital of Fujian Medical University, Fuzhou, Fujian Province People’s Republic of China

**Keywords:** Flexible video endoscope, Macintosh laryngoscope, Tracheal intubation, Lateral decubitus position

## Abstract

**Background:**

Tracheal intubation with the patient in the lateral position is difficult because the laryngeal view is compromised during direct laryngoscopy. Flexible video endoscopes may facilitate intubation even when laryngeal views are poor on direct laryngoscopy because the patients are positioned laterally. Thus, this trial aims to compare the efficacy of flexible video endoscopes to Macintosh laryngoscopes for orotracheal intubation in the lateral position and to investigate their feasibility, i.e., whether the use of the two devices in combination can secure the airway when endotracheal intubation in the lateral position has failed using one device.

**Methods:**

This will be a prospective, randomized, single-center, clinical trial. One hundred and seventy-four patients aged 18–65 years, who have been scheduled to undergo tracheal intubation under uniform general anesthetic techniques for elective kidney surgery in the lateral decubitus position will be randomly divided into the flexible video endoscope and the Macintosh laryngoscope groups. Primary outcomes include intubation time and intubation success rate. Secondary outcomes include overall user satisfaction (graded from 1 to 10 (1 = very poor, 10 = excellent)) and perioperative side effects and complications, such as frequency of esophageal intubation, lip or dental injury, sore throat, and hoarseness.

**Discussion:**

The trial will clarify the efficacy of intubation with a Macintosh laryngoscope and a flexible video endoscope in the lateral position, and whether the two devices could be used in combination to secure the airway in cases where endotracheal intubation in the lateral position has failed with one device.

**Trial registration:**

Chinese Clinical Trial Register, ChiCTR- IOR-15007175. Registered on 6 October 2015.

**Electronic supplementary material:**

The online version of this article (10.1186/s13063-019-3263-1) contains supplementary material, which is available to authorized users.

## Background

In certain situations, patients require emergency endotracheal intubation to maintain airway patency in the lateral position, such as in accidental pulling out of the endotracheal tube during surgery, inadequate regional anesthesia that warrants general anesthesia, and intubation in the presence of bleeding in the airways above the larynx to prevent the risk of aspiration [1, 2]. Sometimes, clinicians may encounter a clinical situation where the supine position is not possible, such as a patient’s inability to lie down in the supine position because of a large lumbar mass, and the airway then has to be secured with the patient in the lateral position [3]. Moreover, patients undergoing surgical procedures in the lateral position may benefit from endotracheal intubation performed in the lateral position because it avoids the risk of adverse events caused by switching an anesthetized patient from the supine to any other position [4, 5]. Direct laryngoscopic intubation, as typical airway management, is more challenging in the lateral position than in the supine position primarily because of the distorted airway anatomy and unfamiliar posture that is necessarily required of the operating medical personnel [6].

When patients undergo gastroscopy under propofol anesthesia in the lateral position, the glottis is easily exposed just before the gastrointestinal endoscope passes through the esophageal inlet. A recent report compared the use of a fiberoptic bronchoscope in the supine and lateral positions, and revealed that endotracheal intubation using a fiberoptic bronchoscope might be an effective and timesaving technique for patients in the lateral position [7]. However, use of the flexible video endoscope and Macintosh laryngoscope have not been compared in the lateral position in prospective trials. The flexible video endoscope used in this clinical trial is very similar in structure and function to a fiberoptic bronchoscope. Our hypothesis is that the differences in construction and operational features of the Macintosh laryngoscope and the flexible video endoscope would result in differences in clinical performance in patients in the lateral position.

In a population of patients with low a priori predicted risk of difficult airway control, we sought to (1) compare the effects of intubation with a Macintosh laryngoscope to the effects of intubation with a flexible video endoscope with the patient in the lateral position and (2) investigate the feasibility of endotracheal intubation by combining the use of the Macintosh laryngoscope and a flexible video endoscope, i.e., determine whether the use of both devices in combination could secure the airway when endotracheal intubation in the lateral position has failed using one device.

## Methods

The study will be conducted at the First Affiliated Hospital of Fujian Medical University (Chinese Clinical Trial Register, ChiCTR- IOR-15007175). The study protocol was prospectively approved by the First Affiliated Hospital of Fujian Medical University Ethics Committee.

One hundred and seventy-four patients with American Society of Anesthesiology (ASA) physical status I–II, aged 18–65 years old, who are scheduled for elective kidney surgery in the lateral decubitus position and require endotracheal intubation for general anesthesia will be enrolled in this study. Patients with a body mass index (BMI) > 30 kg/m^2^, cervical spine abnormality, pharyngolaryngeal disorder, anticipated difficult-to-intubate airway, or increased risk of aspiration will be excluded.

**Fig. 1:**
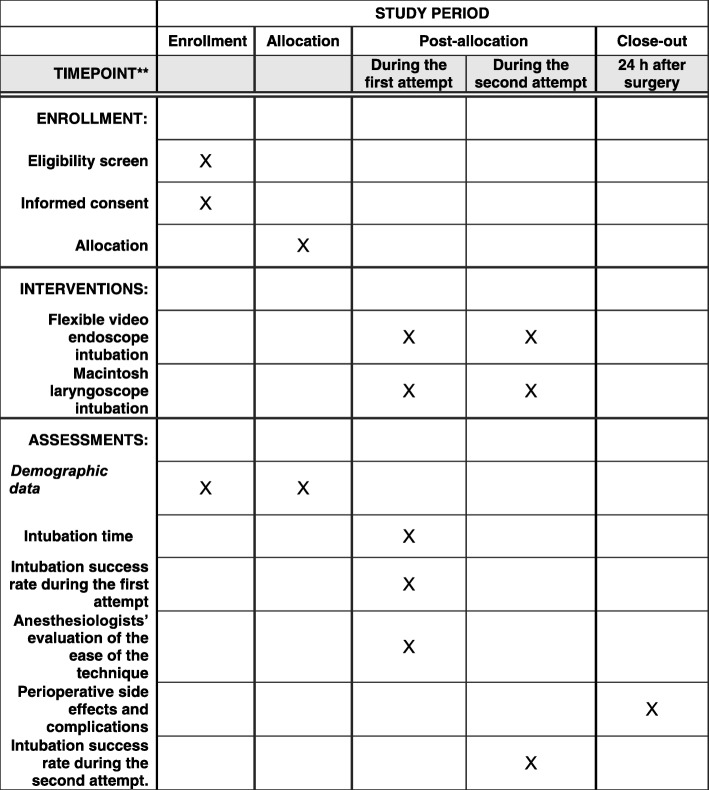
Flow chart of the present study. ASA, American Society of Anesthesiology; BMI, body mass index

Randomization will be performed using computer-generated random numbers, with group and number assignment being stored in sealed envelopes. An envelope will be drawn in the presence of other staff immediately before the patient enters the operating room. Patients will be randomly assigned into two groups to receive flexible video endoscope intubation (FEI) or Macintosh laryngoscope intubation (MLI). Patients within each group will also be randomized to receive intubation in the left or right lateral decubitus position. Figure 1 shows the flow of the patients through the study.

### Protocol

**Fig. 2:**
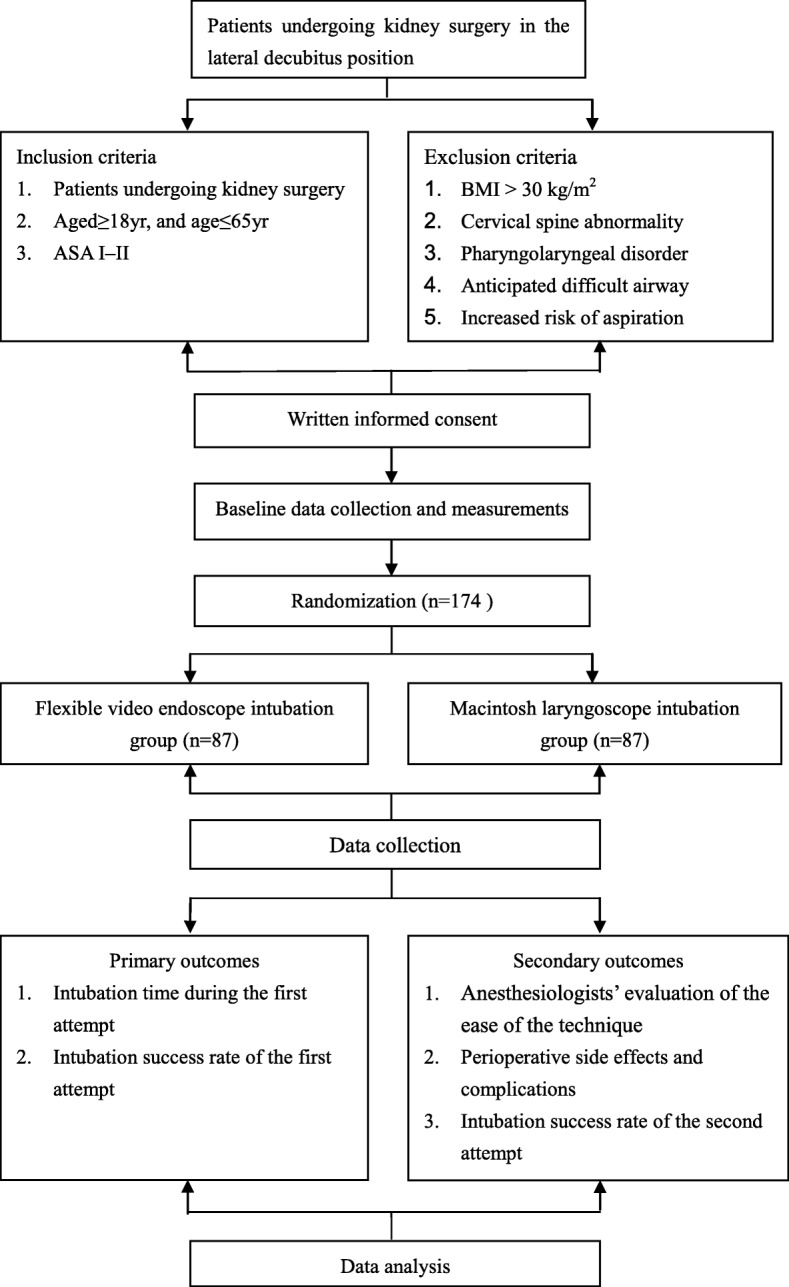
Schedule of enrollment, intervention and assessments

The schedule of enrollment, intervention and assessments is shown in Fig. 2. Patients will have standard heart rate (HR), noninvasive blood pressure, and pulse oximetry monitored in the operating room. Penehyclidine hydrochloride 10–12 μg/kg/body weight (maximum dose 1 mg) will be administered after placement of an intravenous catheter. Subsequently, patients will be positioned in either the right or left lateral decubitus position, determined by the site of the surgical procedure, and the patient’s head will be placed in the sniffing position. A pillow, along with a sheet beneath the underside of the head and neck, will be used to maintain the axial alignment, and both arms will be placed perpendicular to the torso over the armrest to support the weight; this position will be maintained during induction of anesthesia and intubation. Following adequate preoxygenation using 100% oxygen via a face mask for 3 min, anesthesia will be induced via an intravenous injection of 1.5–2.5 mg/kg/body weight of propofol and 2.0–4.0 μg/kg/body weight of fentanyl. Rocuronium (0.6 mg/kg/body weight) will be administered for neuromuscular blockade after confirmation of adequate facemask ventilation. Bag-mask ventilation will be continued for 3 min with 100% oxygen and 2% inspired sevoflurane. Neuromuscular blockade depth will be measured using time of flight (TOF) Watch SX acceleromyography. The patient’s oropharyngeal secretions will be cleared with a suction tube after complete muscle relaxation is confirmed using a nerve stimulator, with a TOF value of 0.

Two anesthetists, who are not informed about the use of the Macintosh laryngoscope or the flexible video endoscope until the patient arrives in the operating room, will perform all intubations. These anesthetists have experience of > 4000 intubations using the Macintosh laryngoscopes and > 400 using the flexible video endoscope with the patient in the supine position. However, as they have fewer such experiences with the patient in the lateral position, they will practice tracheal intubation in this position using a mannequin before study initiation. A mannequin attached to a baseboard is placed in the right or left lateral position by holding the baseboard at 90° to the horizontal plane. The anesthetists will practice until the trachea of this mannequin could be successfully intubated with a Macintosh laryngoscope and a flexible video endoscope in both lateral positions within 60 s.

In the MLI group, an experienced anesthetist will stand at the head of the operating table to perform laryngoscopy using the Macintosh blade in the standard fashion. External laryngeal manipulation and adjustment of the position of the patient’s head and neck will be performed by other medical personnel as necessary. In the FEI group, a bite block will be positioned between the upper and lower incisors. An experienced anesthetist will stand in front of the patient’s head to perform intubation using a flexible video endoscope (Mindhao Medical Technology Co. Ltd., Zhuhai, China) following standard procedures, with manipulation of the mandibular angle by other medical personnel. Head and neck positions will be adjusted as required.

An assistant will measure the time to intubation, which is defined as the time taken from insertion of the Macintosh blade or flexible video endoscope between the teeth until successful intubation is confirmed by detecting end-tidal carbon dioxide [8]. To maximize patient safety, only one intubation attempt will be performed in the lateral position. If the attempt takes longer than 60 s or the tracheal tube is inserted into the esophagus, it will be deemed a failure, which will be recorded as 61 s [2, 8–10]. Upon failure of the initial endotracheal intubation, facemask ventilation will be performed for 3 min, and anesthesia will be maintained using sevoflurane 2% in oxygen.

All cases of failure of the first intubation in both groups will be followed by the combined use of the Macintosh laryngoscope and flexible video endoscope during the second attempt. An assistant will perform laryngoscopy using a Macintosh blade in the standard fashion and will hold the position to expose the epiglottis regardless of whether the glottis is exposed. Meanwhile, another operator will insert a flexible video endoscope into the mouth and intubate the trachea following standard procedures. If the attempt takes longer than 60 s or the tracheal tube is inserted into the esophagus, it will be deemed a failure. In the case of failed intubation during the second attempt, difficult mask ventilation, or occurrence of desaturation, the patient will be immediately returned to the supine position. The schedule of enrollment and assessments is as in the Standard Protocol Items: Recommendations for Interventional Trials (SPIRIT) Figure (Fig. 1 and Additional file 1: SPIRIT checklist).

### Study outcomes

The primary outcome is the intubation success rate. Secondary outcomes include the intubation time, the anesthesiologist’s evaluation of the ease of the technique (using a visual analog scale (VAS) 0–10 [11]), and perioperative side effects and complications. Frequency of esophageal intubation, lip or dental injury, sore throat, and hoarseness will be recorded by a non-blinded observer. Intubation time will be measured using a stopwatch, and each failed intubation attempt will be recorded as 61 s. Secondary outcomes also include the intubation success rate of the combined use of the Macintosh laryngoscope and flexible video endoscope during the second attempt. The patients will be followed up for 24 h after surgery. 

### Statistical analysis

The minimum sample size is estimated on the basis of intubation success rate. Based on previous reports of a 85.3% intubation success rate using the Macintosh laryngoscope with the patient in the lateral position [2] and a 97% intubation success rate using the flexible video endoscope with the patient in the lateral position [7], assuming that 10% of participants would drop out, 87 patients would be required in each group to detect such a difference with a significance level of 5% and power of 80%. PASS 11 (NCSS, LLC; Kaysville, UT, USA) was used for the sample size calculation. Demographic data will be compared using Student’s *t* test. Time to intubation and overall user satisfaction score will be analyzed using the Mann–Whitney U test. Comparison of various rates will be performed using the chi square (χ^2^) test with Yates correction or Fisher’s exact probability test. A *P* value <0.05 is considered statistically significant.

## Discussion

In this clinical trial, we aim to evaluate the efficacy of intubation using a Macintosh laryngoscope or a flexible video endoscope with the patient in the lateral position. Anatomic distortion of the tongue and other tissues of the upper airway differ in the lateral position and supine positions because of the effect of gravity, which can make direct laryngoscopy and subsequent intubation challenging. In addition, endotracheal intubation using direct laryngoscopy in patients in the lateral decubitus position may be difficult in an unfamiliar posture for anesthesiologists and it may take longer to obtain a favorable view of the glottis. Furthermore, even when a grade 1 or 2 laryngoscopic view is attained, it takes longer to confirm successful intubation because the passage of the tracheal tube through the vocal cords is not observed in some patients.

Although fiberoptic bronchoscopy is an important tool to cope with difficult-to-intubate airways, there are only a few studies [7, 12] wherein endotracheal intubation was performed using fiberoptic bronchoscopes with the patient in the lateral position. Li et al. [7] reported that the tracheal intubation success rate with fiberoptic bronchoscopes was significantly higher in the lateral position (97%) compared with that in the supine position (16%), which was contradictory to the results obtained using the Macintosh laryngoscope. Anesthesia administration with the patient in the supine position causes the patient’s tongue and soft tissues of the throat to sag downwards, which can obstruct the operator’s view and the forward motion of a flexible fiberoptic bronchoscope [13]. A possible reason for high success rates using the fiberoptic bronchoscope in the lateral decubitus position might be alleviation of the obstructive effect in this position [7].

Tracheal intubation using a Macintosh laryngoscope, the blade of which is not configured symmetrically, is considered to be easy with the patient in the left lateral position. The difficulty with patients lying in the right lateral decubitus position is the positioning of the tongue, which (influenced by gravity) has a tendency to slip off the laryngoscope blade while the blade is being inserted from the right side of the tongue and moved centrally upwards, thus necessitating shifting the tongue to the left side [14]. The special construction of flexible video endoscopes means that regardless of the patient’s position, anesthesiologists can maintain the upright position to operate. Anesthesiologists can operate flexibly to reveal the glottis by rotating the flexible video endoscope stem and regulating movement of the endoscope tip, which is different from what is possible using a Macintosh laryngoscope in the lateral position.

Another purpose of this clinical trial is to evaluate the effectiveness of the combined use of the Macintosh laryngoscope and flexible video endoscope in difficult airways. In cases where exposure of the glottis is difficult using a Macintosh laryngoscope in the lateral position, the epiglottis can usually be exposed and provoked. Subsequently, a flexible video endoscope can easily help to locate the epiglottis, guided by a Macintosh laryngoscope; thereafter, movements of the front of the flexible video endoscope can be adjusted to locate and enter the glottis.

Our experimental design has certain potential limitations. First, to ensure patient safety, only one attempt with either a Macintosh laryngoscope or a flexible video endoscope will be conducted. If a second or even a third attempt was allowed, the success rate would be different, as the second or the third attempt can also succeed in some cases. Therefore, in the present study, the success rate of tracheal intubation is the success rate only of the first attempted intubation. Second, to maximize patient safety, failed intubation is defined as an intubation attempt that took > 60 s, according to the definition by McCaul et al. [9]. Third, to ensure that the anesthesiologist is adequately skilled in the operation of endotracheal intubation with the patient in the lateral position, the anaesthetist has to practice on a mannequin until the time of intubation is < 60 s. However, this aspect does not reflect real conditions in hospital.

The structure and mode of operation of the Macintosh laryngoscope and the flexible video endoscope are different; therefore, the use of the flexible video endoscope for tracheal intubation with the patient in the lateral position may result in greater success compared with that using a Macintosh laryngoscope. For patients in whom tracheal intubation is difficult in the lateral position, laryngoscopes can be used to increase the space of the pharynx and expose the epiglottis, and subsequently, tracheal intubation can be performed more easily using the flexible video endoscope because of its flexibility of operation.

In conclusion, in this clinical trial, we aim to evaluate the efficacy of intubation using a Macintosh laryngoscope and a flexible video endoscope with the patient in the lateral position and investigate the feasibility of using both devices in combination, when endotracheal intubation in the lateral position has failed with one device. The results of the trial will improve the success rate of tracheal intubation in the lateral position.

## Trial status

Study subjects are currently being recruited into the trial.

## Additional file

Additional file 1: Standard protocol items: recommendation for interventional trials (SPIRIT) 2013 checklist: recommended items to address in a clinical trial protocol and related documents*. (DOC 124 kb)

## Abbreviations

ASA: American Society of Anesthesiology
BMI: Body mass index
FEI: Flexible video endoscope intubation
HR: Heart rate
MLI: Macintosh laryngoscope intubation
